# Paramagnetic Defects and Thermoluminescence in Irradiated Nanostructured Monoclinic Zirconium Dioxide

**DOI:** 10.3390/ma15238624

**Published:** 2022-12-02

**Authors:** Daria V. Ananchenko, Sergey V. Nikiforov, Konstantin V. Sobyanin, Sergey F. Konev, Alma K. Dauletbekova, Gulzhanat Akhmetova-Abdik, Abdirash T. Akilbekov, Anatoli I. Popov

**Affiliations:** 1 Department of Physics and Technology, Ural Federal University, Ekaterinburg 620002, Russia; 2 Department of Technical Physics, L. N. Gumilyov Eurasian National University, Astana 010000, Kazakhstan; 3 Institute of Solid State Physics, University of Latvia, 8 Kengaraga Str., LV-1063 Riga, Latvia

**Keywords:** zirconium dioxide, ion irradiation, electron irradiation, paramagnetic defects, *F^+^* centers

## Abstract

The ESR spectra of nanostructured samples of monoclinic ZrO_2_ irradiated by electrons with energies of 130 keV, 10 MeV, and by a beam of Xe ions (220 MeV) have been studied. It has been established that irradiation of samples with electrons (10 MeV) and ions leads to the formation of radiation-induced *F^+^* centers in them. Thermal destruction of these centers is observed in the temperature range of 375–550 K for electron-irradiated and 500–700 K for ion-irradiated samples. It is shown that the decrease in the concentration of *F^+^* centers is associated with the emptying of traps responsible for thermoluminescence (TL) peaks in the specified temperature range. In the samples irradiated with an ion beam, previously unidentified paramagnetic centers with g = 1.963 and 1.986 were found, the formation of which is likely to involve Zr^3+^ ions and oxygen vacancies. Thermal destruction of these centers occurs in the temperature range from 500 to 873 K.

## 1. Introduction

Zirconium dioxide is a wide-gap dielectric (E_g_ = 5–7 eV), which has high mechanical (strength, refractoriness, corrosion resistance), as well as functional (transparency in a wide range of wavelengths, high refractive index, high ionic conductivity) properties. These properties determine the wide possibilities of using ZrO_2_ in industry as a material for electrochemical devices, and oxygen sensors [1,2,3,4], as well as materials for photocatalysis [5,6,7,8,9] and optical devices [10,11,12,13,14,15,16,17]. The optical and luminescent properties of this material are largely determined by the presence of structural defects, which is especially important when it is used in conditions of harsh radiation environments (nuclear power plants, space electronics). Corpuscular radiation with a particle energy above the threshold (>0.3 MeV for electrons) causes the formation of *F*-type centers (oxygen vacancies with trapped electrons) in ZrO_2_ and similar compounds by the impact mechanism [18,19,20,21]. It is known that these defects have a significant effect on the optical and luminescent properties of binary and complex wide-band gap oxide dielectrics [20,21,22,23,24,25,26].

One of the most sensitive methods for studying radiation-induced defects in materials is electron spin resonance (ESR) spectroscopy. The main paramagnetic defects in ZrO_2_ are centers associated with Zr^3+^ ions (surface and bulk Zr^3+^, *T*-center) [27,28,29,30,31], oxygen hole-centers or OHCs (O^−^ centers) [28,32], and *F-*type (singly ionized oxygen vacancies (*F^+^*) and divacancies (*F_2_^+^*)) [27,28,30,33].

Despite the sufficient number of publications on the topic of paramagnetic radiation defects in ZrO_2_, their thermal properties remain insufficiently studied. The study of the thermal stability of radiation defects is an important problem, since thermal annealing is a way to eliminate defects and restore the performance characteristics of devices based on ZrO_2_. The thermal decay of paramagnetic centers can be accompanied by the emission of TL peaks. It is known that the TL curve of ZrO_2_ irradiated with electrons contains two peaks at 400 and 500 K [34,35]. However, the relationship between these TL peaks and the decay of radiation-induced paramagnetic defects in ZrO_2_ remains unclear.

The study of the relationship between paramagnetic defects and TL properties of the nanostructural modification of zirconium dioxide is of particular interest. It is known that nanostructured materials have an increased resistance to the formation of radiation defects in comparison with single-crystal analogs, and therefore they are promising materials for high-dose (more than 10 Gy) luminescent dosimetry.

The purpose of this work is to identify paramagnetic defects in samples of monoclinic nanostructured ZrO_2_ irradiated with heavy ions and electrons of various energies and to evaluate their thermal stability. It is important to note here that the thermal stability of paramagnetic defects and their relationship to TL properties in monoclinic ZrO2 have not been previously studied.

## 2. Materials and Methods

Samples in the form of tablets with a diameter of 5 mm and a thickness of 1 mm were prepared by cold uniaxial pressing from a nanopowder with a nanoparticle size of 30–50 nm provided by the Plasmotherm Company ((Moscow, Russia). The nanopowder was produced by plasma synthesis; ZrCl_4_ was used as a raw material for its production. Characterization of the pellets was carried out by the SEM method. The SEM image (Figure S1) and particle size distribution (Figure S2) for ZrO_2_ pellets are given in the Supplementary Materials. The average particle size in the pellets, determined by the SEM method, corresponds to the one declared by the manufacturer. The proportion of monoclinic ZrO_2_ in the powder under study, according to X-ray fluorescence data, was at least 99.02%; HfO_2_ (0.331%), Cl (0.458%), K_2_O (0.110%), TiO_2_ (0.069%), PdO (0.009%), and Rb_2_O (0.006%) compounds were present as impurities [36].

The studied samples were irradiated with three different types of radiation: (1) xenon ions with an energy of 220 MeV at the DC-60 heavy ion accelerator (Nur-Sultan, Kazakhstan); (2) electrons with an energy of 130 keV from the pulsed electron accelerator RADAN (pulse duration 2 ns, current density 60 A/cm^2^); (3) electrons with an energy of 10 MeV from the linear electron accelerator UELR (Yekaterinburg, Russia). Two types of electron beams with different electron energies were used, since one of them (10 MeV) is capable of leading to the formation of new F-type radiation centers by the impact mechanism, and the second (130 keV) only changes the charge states of existing defects or impurities. The displacement energies for ZrO_2_ under ion irradiation were estimated using the method presented in [37,38]. Damage was modeled using the SRIM Pro 2013 software code [39] with threshold displacement energies of 40 eV for both zirconium and oxygen atoms. For fluences from 10^9^ to 10^13^ ion/cm^2^, the corresponding dpa values ranged from 0.5 × 10^−7^ to 0.5 × 10^−3^ dpa. Evaluation of dpa upon irradiation with 10 MeV electrons is carried out according to the formula presented in [40]. The corresponding value of the atomic displacement cross section was taken from [41]. According to the calculated value of the displacements, the fluence of 10^15^ electrons/cm^2^ is 1.3 × 10^−6^ dpa.

TL was measured with a photomultiplier tube with a spectral sensitivity maximum at 400–420 nm under a linear heating rate of 2 °C/s.

ESR measurements were performed on a Bruker ELEXSYS 580 device with a resonant frequency of 9.78 GHz in the range of the magnetic flux density change from 50 to 600 mT. ESR spectra were recorded at room temperature after heating the samples in the range of 323–873 K with a step of 20 K. When studying the thermal stability of paramagnetic centers, the samples were heated in the same mode as in the measurement of TL.

## 3. Results

**Figure 1:**
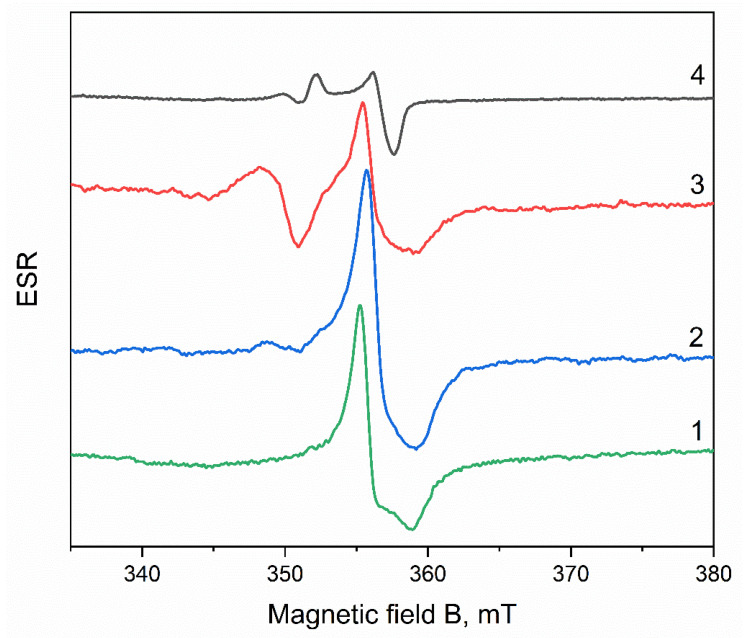
ESR spectra of unirradiated ZrO_2_ (1) and irradiated with an electron beam with an energy of 130 keV (dose 15 kGy) (2), 10 MeV (fluence 10^15^ electrons/cm^2^) (3), and Xe ions with an energy of 220 MeV (fluence 10^13^ ions/cm^2^) (4).

To identify radiation-induced defects in ZrO_2_, the ESR spectra of the initial ZrO_2_ pellets irradiated with various radiation sources were measured (Figure 1). The ESR spectrum of unirradiated ZrO_2_ pellets contains a signal at a magnetic field B = 356.0 mT with g = 1.965 and a peak-to-peak width 3.7 mT. This signal is associated with paramagnetic Zr^3+^ ions, which were probably formed during the synthesis of the initial ZrO_2_ nanopowders. The ESR signal of Zr^3+^ ions has already been detected earlier in unirradiated ZrO_2_ [27,42] and ZrO_2_:Er [28] nanopowders. The signal with g_⊥_ = 1.975 and g_II_ = 1.957–1.958 was identified in them as bulk Zr^3+^. In [28,29], an ESR signal with identical g_⊥_ but different g_II_ = 1.905–1.928 was assigned to surface Zr^3+^ ions. The g factor of the paramagnetic Zr^3+^ found by us in the initial pellets makes it possible to identify it as bulk Zr^3+^.

In the ESR spectra of the samples irradiated with electrons with an energy of 130 keV (a dose of 15 kGy), as in the initial pellets, there is a signal from Zr^3+^ with g = 1.965 and a peak-to-peak width of 3.2 mT. In addition, a low-intensity signal with g = 1.999 appears. This signal indicates the presence of a trace concentration of *F^+^* centers in the irradiated samples. Previously, the *F^+^*-center was detected in ZrO^2^ after electron and ion irradiation and represented a line with a small g-factor anisotropy (g_⊥_ = 1.972 and g_II_ = 1.996) [40]. Some authors report that the *F^+^* center is characterized by an isotropic signal with a g-factor near the free electron value (g_e_ = 2.0023) [27,28]. Previously, the formation of F^+^ centers upon irradiation with an electron beam with an energy of 130 keV can occur because of the capture of electrons by oxygen vacancies present in the initial samples, and in the photoluminescence (PL) and pulsed cathodoluminescence (PCL) spectra of the samples studied in this work. A luminescence band was observed at 480 nm, in which the formation of oxygen vacancies participate [35].

Irradiation of the pellets with electrons with an energy of 10 MeV (Figure 1) leads to an increase in the intensity of the ESR signal with g = 1.999 (peak-to-peak width 2.7 mT) from *F^+^*-centers. This indicates intensive processes of generation of these centers in ZrO_2_ by the impact mechanism. It is known that the threshold energy for the formation of *F*-type defects in ZrO_2_ is about 1 MeV [18]; therefore, an electron energy of 10 MeV is sufficient to displace oxygen atoms from the lattice sites. The intensity of the ESR signal of Zr^3+^ in samples irradiated with electrons with an energy of 10 MeV is lower than in the initial samples. Additional studies are required to elucidate the cause of this effect.

Irradiation of the investigated ZrO_2_ samples with xenon ions with a fluence of up to 10^13^ ions/cm^2^ did not lead to a change in the ESR spectrum compared to the initial sample. The spectra of these pellets contain a Zr^3+^ signal with g = 1.963 (peak-to-peak width 3.5 mT). In samples irradiated with an ion beam with a fluence of 10^13^ ions/cm^2^, the ESR spectrum is significantly transformed, and the signal from Zr^3+^ ions is no longer observed in it (Figure 1). Instead, signals appear at 356.8 mT (g = 1.963), 350.0 mT (g = 1.998), and 352.5 mT (g = 1.986). The signal with g = 1.998 (peak-to-peak width 1.2 mT) is probably associated with the presence *F^+^* centers in samples irradiated with ions. Signals with g = 1.986 and 1.963 (peak-to-peak width 1.5 mT) can be attributed to a new previously unidentified radiation-induced center.

It is well known that ion irradiation can quite often lead to material amorphization. Previously, the authors of [43] found that the amorphization of nanocrystalline monoclinic ZrO_2_ is possible upon irradiation with uranium ^238^U and Au ions with energies above 1 GeV, when the energy losses exceed 40.2 keV/nm. It was shown in [44] that amorphization of ZrO_2_ irradiated with xenon ions with energies of 300–400 keV occurs at a peak displacement damage level of about 680 dpa. We have estimated the appearance of an amorphous phase in the studied ZrO_2_ pellets upon irradiation with Xe ions of 220 MeV energy. To do this, using the SRIM program, the values of electronic energy losses and displacement per atom were calculated. The calculated value of electronic energy losses were found to be 19.8 keV/nm, which is less than the energy losses at which ZrO_2_ amorphization begins. The value of displacements for fluences from 10^9^ to 10^13^ ions/cm^2^ was from 0.5 × 10^−7^ to 0.5 × 10^−3^ dpa, respectively, which is also significantly less than dpa, at which ZrO_2_ amorphization was observed in [44]. Considering the above, we can conclude that in the samples we studied, the formation of an amorphous phase is negligible.

**Figure 2:**
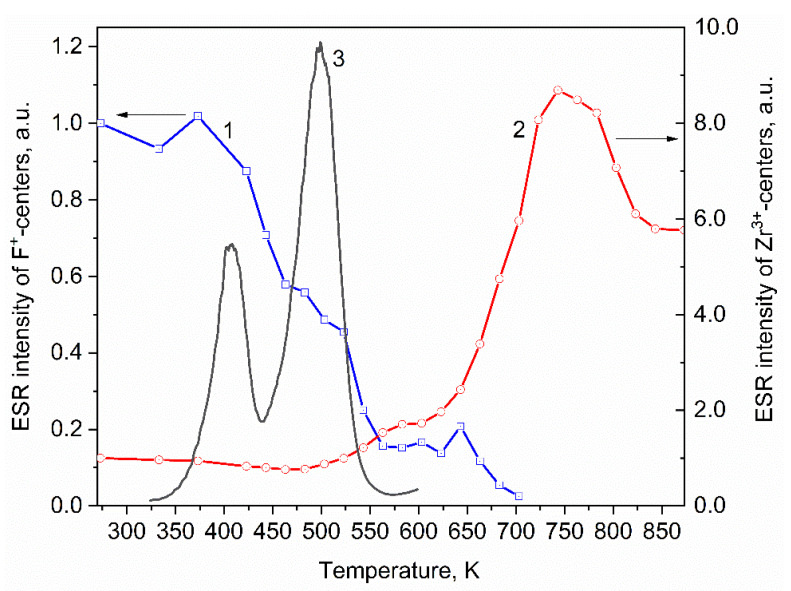
Dependence of the intensity of the ESR signal of *F^+^*-centers (1), Zr^3+^ ions (2) on the heating temperature and TL (3) of ZrO_2_ irradiated with an electron beam with an energy of 10 MeV.

The thermal stability of paramagnetic *F^+^* centers (g = 1.999) and Zr^3+^ ions (g = 1.965) in pellets irradiated with 10 MeV electrons was studied. Let us consider the temperature dependence of the ESR intensity for the *F^+^*-center (Figure 2). The intensity of this signal decreases at temperatures above 375 K. A sharp drop in intensity is observed in the temperature range from 375 to 550 K. This temperature range coincides with the temperatures at which TL is observed in the samples under study. Figure 2 shows that the TL curve of samples irradiated with 10 MeV electrons contains two TL peaks at 410 and 500 K. Since the heating of the samples in the study of the thermal stability of paramagnetic centers and the measurement of TL was carried out in the same mode, it can be concluded that the change in the concentration of *F^+^* centers during heating can be associated with a change in their charge state due to the capture of electrons released from the traps responsible for TL. Previously, in [35], based on the results of studying the processes of TL quenching in monoclinic ZrO_2_, the electronic nature of the TL peaks at 390 and 485 K was assumed.

The thermal stability of radiation-induced *F^+^* centers was studied in [45,46,47]. In a parallel study of TL and ESR, the authors suggested that the monotonic decrease in the concentration of *F^+^*-centers to 550–600 K may be due to its transition to the diamagnetic state (*F* center) as the result of the capture of an electron released from the trap. However, two results contradicted this assumption. First, the temperature of the maximum found on the TL curve (550 K) exceeded the temperature at which 50% of the *F^+^*-centers disappear (450 K). Second, the calculated value of the trap depth responsible for the TL peak at 550 K, obtained by the initial rise method, was 0.7–1.1 eV, which is somewhat larger than the defect annealing activation energy of 0.3–0.7 eV calculated from ESR data. The results obtained by us (Figure 2) clearly indicate the relationship between the decrease in the intensity of the ESR signal of *F^+^*-centers at T = 375–550 K and the emission of TL peaks in the indicated temperature range, which confirms the assumption made by the authors of [45,46,47]. It is well known that an alternative mechanism for annealing radiation-induced *F*-type defects in oxides is the recombination of oxygen vacancies with interstitial oxygen, the diffusion of which becomes possible when the crystal is heated [48,49,50]. Previously, the authors of [51] showed that in zirconium oxide irradiated with neutrons (E > 0.1 MeV), the diffusion of interstitial oxygen becomes possible at temperatures above 500–600 K. In the samples studied by us, a decrease in the concentration of *F^+^* centers is observed at lower temperatures of 375–550 K, which testifies in favor of the proposed mechanism for the destruction of *F^+^* centers associated with their ability to capture the charge carriers released from traps that cause TL peaks.

**Figure 3:**
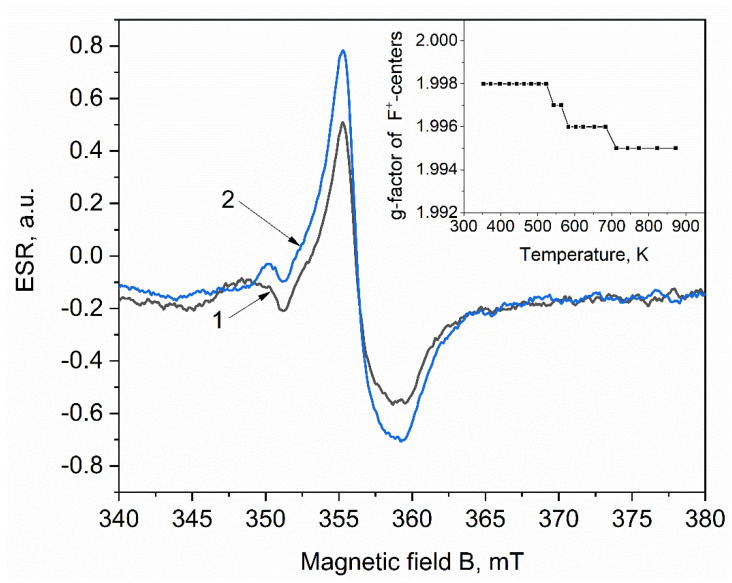
ESR spectra of a ZrO_2_ pellet irradiated with electrons with an energy of 10 MeV, and then annealed at temperatures of 540 K (1) and 580 K (2).

It should also be noted that at temperatures of 550–700 K, the decrease in the concentration of F^+^ centers slows down and a change in the shape of the ESR signal is observed (Figure 3). More detailed studies have shown that at T = 525–700 K, the g-factor of the *F^+^*-center shifts from 1.998 to 1.995 (Figure 3 inset). The shift of the g-factor of the *F^+^* center may indicate a change in its local crystalline environment. This assumption can be supported by an increase in the ESR intensity of Zr^3+^ ions in the same temperature range. In this case, the Zr^3+^ and ions can change the local environment of the *F^+^* centers.

The thermal stability of Zr^3+^ ions has also been studied. Figure 2 shows that in the temperature range of 280–525 K, the intensity of the ESR signal associated with Zr^3+^ does not change. With a further increase in temperature to 750 K, it increases by a factor of 8.7. At temperatures above 750 K, the signal intensity drops, but does not return to the value characteristic of the irradiated sample. The change in the ESR intensity of Zr^3+^ is not associated with the emptying of traps in the material under study, since, according to our experiments, no TL peaks were observed in the temperature range of 600–850 K. At the same time, the values of its intensity did not exceed the background values caused by the thermal radiation of the heating element.

An increase in the Zr^3+^ concentration with an increase in the annealing temperature was observed earlier in [52]. The authors associated it with the transformation of Zr^4+^ ions into Zr^3+^ caused by electron capture. In this case, the electron donors can be oxygen vacancies that have captured one or two electrons, as well as O^−^ ions if there is a deficiency of positive charge in the cationic sites located near these ions. These processes can also take place in the samples studied in this work, since they contain defects associated with oxygen vacancies.

**Figure 4:**
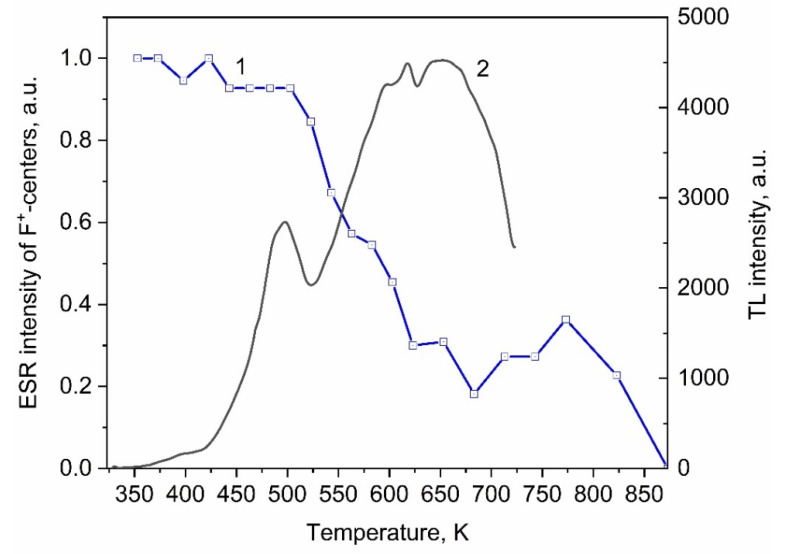
Dependence of the intensity of the ESR signal of F^+^-centers on the heating temperature (1) and TL (2) of ZrO_2_ samples irradiated with xenon ions.

Next, we studied the thermal stability of ESR signals in samples irradiated with ions. Figure 4 shows the dependence of the intensity of the ESR signal of *F^+^* centers (g = 1.998) on the heating temperature in ZrO_2_ samples irradiated with a xenon ion beam with a fluence of 10^13^ ions/cm^2^. The intensity of this signal begins to decrease at temperatures above 500 K, which is 125 K more than in the samples irradiated with electrons. Further, the ESR intensity of the *F^+^*-centers monotonically drops to 675 K. In contrast to samples irradiated with electrons, the TL peak at 410 K in ion-irradiated samples has a low intensity. The difference in the temperature at which the annealing of *F^+^* centers begins in the samples irradiated with ions and electrons can be due to the difference in the intensity of this peak. Thermal emptying of the trap responsible for the low-intensity TL peak in ion-irradiated samples does not significantly affect the concentration of *F^+^* centers. The decrease in the concentration of *F^+^-*centers in ion-irradiated samples begins when the trap responsible for the intense TL peak at 500 K is empty. This fact confirms the assumption that the thermal decay of *F^+^* centers is related to the TL properties of ZrO_2_.

The data in Figure 4 show that the decrease in concentration *F^+^* centers in the temperature range of 500–700 K is replaced by its growth at higher heating temperatures, which was not observed in the samples irradiated with electrons. The interval of increase in the concentration of *F^+^* centers can also be related to the emptying of traps. The TL curve of samples irradiated with ions, in addition to the peaks at 410 and 500 K observed after electron irradiation, contains an additional peak of a complex shape at 550–750 K, which can contain both electronic and hole components. In this case, the emptying of electron traps will contribute to a decrease in the concentration of *F^+^* centers, while the emptying of hole traps will contribute to their growth, which is observed at T > 700 K. An increase in the concentration of *F^+^* centers upon the emptying of hole traps occurs as a result of the capture of holes by *F* centers. A more thorough study of the TL properties at temperatures above 725 K is required to prove this assumption; however, the measurement of TL at such temperatures is hampered by instrumental limitations, as well as by the presence of an intense thermal background of the heating element.

**Figure 5:**
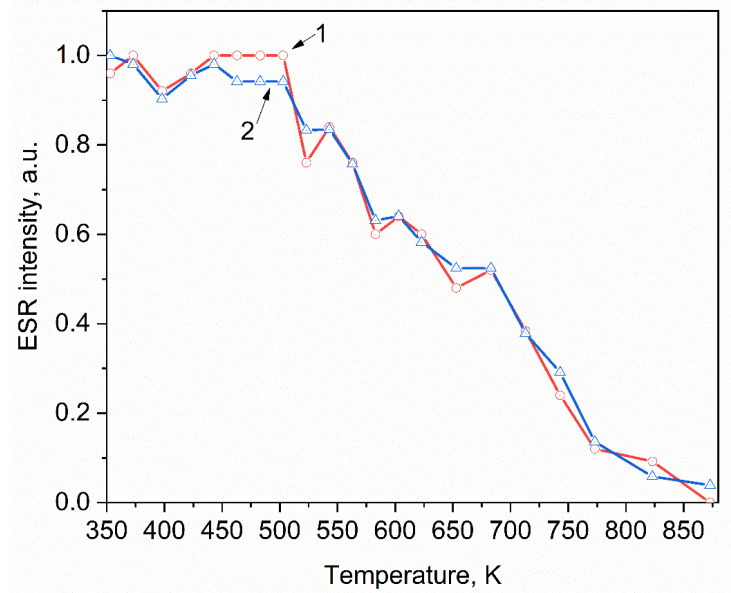
Dependence of the intensity of ESR signals with g = 1.986 (1) and g = 1.963 (2) on the heating temperature of ZrO_2_ samples irradiated with xenon ions.

The thermal stability of paramagnetic signals of an unidentified nature with g = 1.986 and 1.963 (Figure 1) was also studied in pellets irradiated with xenon ions (Figure 5). The temperature dependence of the ESR intensity of these two signals is identical and decreases monotonically to an undetectable level in the temperature range from 500 to 873 K. The identity of the behavior of the signal intensities with g = 1.986 and 1.963 with a change in the heating temperature shows that these signals refer to one paramagnetic center. The nature of this center is probably associated with a complex defect, which includes paramagnetic Zr^3+^ ions and oxygen vacancies. The participation of Zr^3+^ ions in defect formation is indicated by the simultaneous disappearance of the ESR signal of these ions and the appearance of signals with g = 1.986 and 1.963 in ZrO_2_ in pellets irradiated with an ion beam with a fluence of 10^13^ ions/cm^2^. It is also known that when ZrO_2_ is irradiated with ions with an energy above the threshold, an intense generation of anion vacancies occurs in it, which can also contribute to the formation of complex defects [40,53,54]. In the past few years, a large number of such complex defects have been studied in detail in MgO, Al_2_O_3_, and Gd_3_Ga_5_O_12_ crystals [55,56,57,58,59,60].

## 4. Conclusions

1. The formation of radiation-induced F^+^ centers in nanostructured monoclinic ZrO_2_ pellets under irradiation with fast electrons (10 MeV, fluence 10^15^ ion/cm^2^) and Xe ions (220 MeV, fluence 10^13^ ion/cm^2^) has been observed.
2. It was found that the decrease in the concentration of F^+^ centers upon thermal annealing is due to the emptying of traps responsible for TL.
3. However, the change in the ESR intensity of Zr^3+^ centers during thermal annealing does not correlate with the TL of the studied material.
4. Furthermore, irradiation of ZrO2 with Xe ions with a fluence of 10^13^ ions/cm^2^ leads to the appearance of new EPR signals with g = 1.963 and 1.986 being of an unknown nature and stable up to T = 873 K.

## Data Availability

The data presented in this study are available on request from the corresponding author. The data are not publicly available due to the ongoing research.

## Supplementary Materials

The following supporting information can be downloaded at: https://www.mdpi.com/article/10.3390/ma15238624/s1, Figures S1 and S2: SEM and particle size distribution data.
