# Supplementary material for: Paramagnetic Defects and Thermoluminescence in Irradiated Nanostructured Monoclinic Zirconium Dioxide
Source: Materials (Basel). 2022 Dec 2;15(23):8624. doi: 10.3390/ma15238624 (PMC9735659; doi:10.3390/ma15238624)
Supplement: Supplementary file 1 [file materials-15-08624-s001.zip › materials-1979604-supplementary.pdf]

Supplementary. SEM and particle size distribution data.

Figure S1 shows scanning electrom microscope (SEM) image of ZrO_2 surface pellets. It could be seen that the pellets have a porous structure. The shape of the particles is far from spherical due to the adhesion of several particles into larger ones.

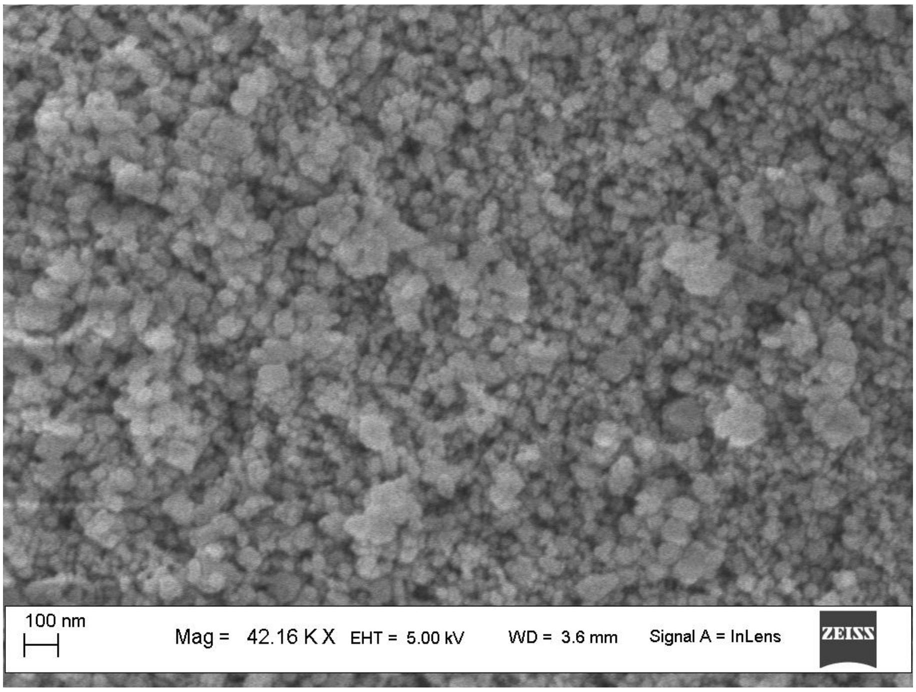

Figure S1. SEM image of ZrO_2 pellets

The histogram of the particle size distribution was constructed based on the SEM results (Figure S2). The particle size was defined as the equivalent diameter equal to the arithmetic mean between the lengths of two mutually perpendicular segments. It can be seen that the mean particle size on the surface of ZrO_2 pellets is 30-50 nm.

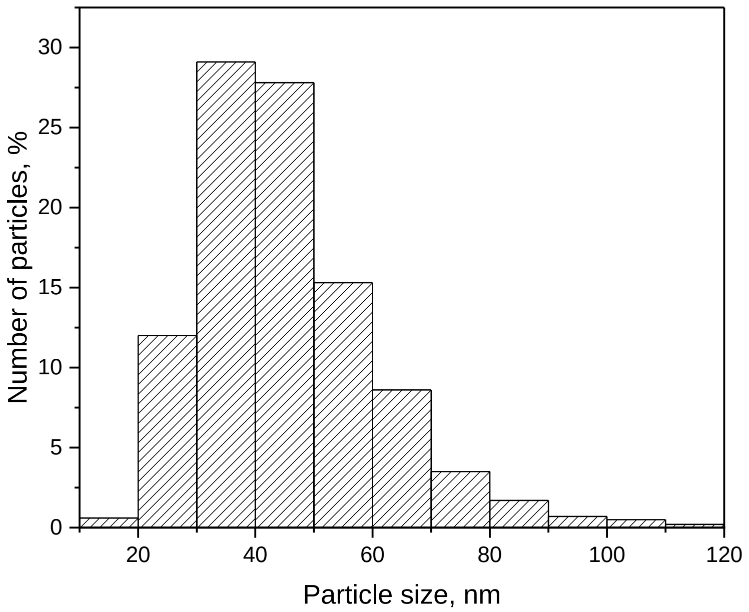

Figure S2. Particle size distribution for the ZrO_2 pellets
